# Neuromedin B Induces Acute Itch in Mice via the Activation of Peripheral Sensory Neurons

**DOI:** 10.2340/00015555-3143

**Published:** 2019-05-01

**Authors:** Sarah EHLING, Tomoki FUKUYAMA, Mei-Chuan KO, Thierry OLIVRY, Wolfgang BÄUMER

**Affiliations:** 1 Department of Molecular Biomedical Sciences, College of Veterinary Medicine, North Carolina State University, North Carolina, USA,; 2 Institute of Pharmacology and Toxicology, Faculty of Veterinary Medicine, Freie Universität Berlin, Germany,; 3 Department of Physiology and Pharmacology, Wake Forest University School of Medicine, Winston-Salem, North Carolina,; 4 Department of Clinical Sciences, College of Veterinary Medicine,; 5 Comparative Medicine Institute, North Carolina State University, North Carolina, USA

**Keywords:** acute itch, dorsal root ganglia, mast cells, neuromedin B, allergic dermatitis

## Abstract

Neuromedin B is expressed in nociceptive and itch-sensitive dorsal root ganglia neurons, but its peripheral pruritogenic potential is not well described. The potential of neuromedin B as a pruritogen and pro-inflammatory peptide in the skin was tested *in vivo* in an acute model in mice and monkeys as well as an allergic dermatitis model in mice. To identify the underlying mechanisms *in vitro* real time PCR analysis for neuromedin B and its receptor expression in murine mast cells and dorsal root ganglia as well as functional calcium imaging in the ganglia was applied. Neuromedin B induces itch when injected intradermally, and the peripheral signal is likely transmitted through the activation of dorsal root ganglia. Thus, neuromedin B could be an interesting new therapeutic target for peripheral processing of itch at the level of sensory neurons.

A cute pruritus (itch) is a protective alarm signal that induces a scratch response to remove hazards from the skin, such as toxins or parasites. Dermatoses were the 18^th^ leading cause of health burden worldwide assessed by the Global Burden of Skin Disease in 2010 (1). Itch is a symptom of a variety of inflammatory skin conditions such as atopic dermatitis (AD) or psoriasis. Particularly chronic itch is a treatment challenge in dermatology (2). Therefore, there is an urgent need for finding strategies to control itch more efficiently that requires a better understanding of the underlying itch and scratch mechanisms.

Pruritus can have its origin directly in the periphery (skin), or it can develop in the central nervous system (CNS) via haematogenic or neurogenic mediators (2, 3). Pruritogenic mediators such as cytokines, proteases, leukotrienes and histamine activate itch-sensing nerve endings in the skin (4, 5). In the last decade, animal models helped to further understand the transmission of the itch signal from the periphery to the CNS and identified pruritogens and their receptors. It is known, that mast cells are located in close proximity to sensory nerve endings in the skin and that they communicate through an intercellular network that includes histamine, tryptase, neuropeptides, leukotrienes, prostaglandin D2, cytokines and chemokines (6). Among these neuropeptides is the mammalian bombesin-like peptide family and its members neuromedin B (NMB) and gastrin-releasing peptide (GRP). The C-terminal amidated sequence of NMB, Leu-Trp-Ala-Thr-Gly-His-Phe-Met-NH_2_, is highly conserved across mammalian species. Neuromedin B is found in the pituitary gland, pancreas, adrenal medulla, gastrointestinal tract and the central nervous system (CNS) (7).

In the CNS, NMB induces scratching when injected intrathecally in mice or intracerebroventrically in rats (8, 9). Neuromedin B is also expressed in peripheral nerves, the trigeminal ganglion and the dorsal root ganglia (DRG). In the latter, NMB is located in nociceptive and itch-sensitive neurons. If injected into the plantar area of the foot in mice, it induces neurogenic inflammation and thermal nociception (10). Neuromedin B signals exclusively through its receptor NMBR, a G protein-coupled receptor (11). This receptor is expressed on cells of the immune and endocrine systems, urogenital and respiratory tracts and the CNS (11). Mishra et al. (10) also showed that NMBR is expressed on peripheral nerves, the trigeminal and DRG, where it is mostly co-expressed with the transient receptor potential cation channel subfamily V member 1 (TRPV1)+ and calcitonin gene-related peptide (CGRP)+ neurons.

Interestingly, recent studies using microarray-based transcriptome profiles, identified an upregulation of NMB in the atopic skin in dogs, which is of importance as dogs are a translational model for human AD (12). In a canine model for acute AD skin lesions, NMB was upregulated 2–3 fold in acute skin lesions 24 and 48 h after allergen challenge in sensitized dogs (13). Corresponding to this, Plager et al. (14) found that NMB was upregulated 2-fold in acute lesional skin of atopic dogs. To our knowledge, whether or not NMB can induce itch peripherally has not been reported.

The first objective of this study was to determine the potential of NMB as a pruritogen and pro-inflammatory peptide in the skin *in vivo*. The second objective was to identify the possible mechanism of NMB to induce acute itch, which could be either via the direct activation of sensory neurons or indirectly via the degranulation of mast cells *in vitro*.

## MATERIAL AND METHODS

### Subjects

#### Mice.

BALB/cAnNCrl mice (BALB/c mice; 5–10-week-old, males and females, 25–30 g) were obtained from Charles River Laboratories (Strain Code 028, Raleigh, NC). Mast cell deficient mice (*Kit*^W-sh^/HNihrJaeBsmJ, stock number 005051, 8-week-old, 2 females, 4 males, 25–30 g) were compared to C57BL/6J mice (stock 000664, The Jackson Laboratory; 11-week-old, 3 females, 4 males, 28–32g). All mice were healthy and housed in groups of 4 or fewer mice per cage in a specific pathogen free environment (sentinel animals, Allentown IVC cages) with bedding (Anderson Bed-o’ Cobs 1/4”) and nesting material (Ancare Nestlets). Room temperature was kept at 22 ± 2°C) with a 12-h light/dark cycle and 50% humidity. Water and a standard diet (LabDiet Rodent Diet 5001, Southern Agriculture) were available *ad libitum*. Environmental enrichment was provided in the home cage. *In vivo* studies were performed following the Institutional Animal Care and Use Committee (IACUC) of the North Carolina State University.

#### Monkeys.

Five adult male and female rhesus monkeys (*Macaca mulatta*), 10–18 years, 6.5–13.5 kg, were kept at an indoor facility accredited by the AAALAC. Animals were individually housed in species-specific rooms with 21–25°C, 40–60% relative humidity and a 12-h light-dark cycle. Their daily diet consisted of 20–28 biscuits (Purina Monkey Chow; Ralston Purina Co., St. Louis, MO), fresh fruit and water *ad libitum*. Primate treats and cage-enrichment devices were supplied. Experiments were approved by the IACUC of Wake Forest University and Wake Forest University School of Medicine. This study is reported in accordance with the ARRIVE guidelines for reporting experiments involving animals (15).

### Acute itch and inflammation model in mice

The rostral back of the mice was clipped (area of 2 × 2 cm) two days before the experiment. The day of the experiment, one or two mice were placed in the experimental home cage. After 30 min habituation time, 50 μl vehicle or NMB were injected intradermally and the mice were video-monitored for one hour. A scratching bout was defined as lifting the hind leg, repeated strokes in the area of injection, and then either placing the paw back on the ground or licking it. For analysis, 10-min intervals were counted as time unit. To determine if NMB could induce acute inflammation, the left pinnae were injected with vehicle (10 μl i.d.) and the right pinnae injected with NMB (10 μg/10 μl). The thickness of the pinnae was determined 1, 3, 6, 9, 24 and 48 h after injection using a spring-loaded thickness micrometer gauge (Mitutoyo, Kanagawa, Japan).

### Allergic dermatitis (TDI-induced) itch and inflammation model in mice

The sensitization in the toluene-diisocyanate (TDI-) model for allergic contact dermatitis was performed as recently published (16). In short mice get sensitized by 5% TDI (in acetone) applied abdominally on 3 consecutive days. On day 21 the reaction is boosted by 0.5% TDI onto the abdomen and on day 28 0.5% TDI is administered onto the ear pinna (challenge phase). To determine the impact on inflammation during the challenge phase, the left pinnae were injected with vehicle (10 μl i.d.) and the right pinnae with NMB (10 μg/10 μl i.d.). Both ears were treated with 10 μl TDI 0.5% topically. The thickness of the pinnae was determined 7 and 24 h after injection using a spring-loaded thickness micrometer gauge (Mitutoyo, Kanagawa, Japan).

To determine the influence on itch, 50 μl vehicle or NMB (50 μg/50 μl) were injected intradermally into the clipped skin area, 30 μl TDI 0.5% was immediately applied topically and the mice were video-monitored for one hour. Scratching bouts were analyzed as described above.

### Acute itch model in monkeys

Monkeys were seated in primate chairs and both lateral sides of the upper part (i.e., the skin area over the *vastus lateralis* muscle) of both hindlimbs were shaved. Shaving and marking the injection site were always conducted at least one day before the test session. The solution of NMB was prepared on the testing day and cleaned with an alcohol swab. The monkey’s hindlimb was held tight by another experimenter before and during the injection. The test agent (vehicle or NMB) in 20 μl solution was slowly injected (50 μl microsyringe, Hamilton Co., Reno, NV) and was watched for wheal to appear. Once the injection was completed, the monkey was immediately returned to his/her home cage and was video-recorded for the scratching activity. Before collecting data, monkeys had been habituated with this injection procedure and the investigator also practiced several times this injection procedure with monkeys beforehand. The injection site was rotated randomly from each site (right vs. left) and each spot (front vs. back) (i.e., RF/RB and LF/LB).

### Murine dorsal root ganglia cell culture and single cell calcium imaging

The isolation of the murine DRG neurons from BALB/c mice was performed, as recently published (17). Mice were euthanized with CO_2_ and immediately the DRG ganglia along the spinal cord (cervical to sacral). Changes in intracellular Ca^2+^ in single cells were measured by digital microscopy connected to equipment for ratiometric recording of single cells, as described previously (17). Ganglia neurons were exposed to NMB (1 mM) or the NMBR antagonist (PD168368 10 μM, 1 min before NMB activation) and the positive control KCL (150 mM) with at least 2 min washing period between stimulations.

### Murine bone marrow-derived mast cells and beta-hexosaminidase degranulation assay

Murine bone marrow cells were harvested from the femur under sterile conditions and 2.5 × 10^6^ cells were cultured in 10 ml media (RPMI 1640, FCS 10%, Pen-Strep 1%, 50 μmol/l beta-mercaptoethanol) and 30 ng/ml murine IL-3 in tissue culture dish cell+ (100 mm × 20 mm, Sarstedt, Nümbrecht). Fifty percent of the media was changed twice per week and cells remained in culture for 6 weeks. Toluidine blue staining was performed to ensure the presence of over 80% of mature mast cells. Mast cell degranulation was determined following a protocol published by Kuehn et al. (18). Murine bone marrow-derived mast cells (BMMC) were stimulated with vehicle, NMB (1 μg/ml or 10 μg/ml), NMBR antagonist (PD168368 4.88 μg/ml, 30 min before NMB activation) or the MrgprB2 agonist mast cell secretagogue compound 48/80 (100 μg/ml). 100 μl PNAG buffer were mixed with 50 μl sample per 96-well and incubated for 90 min at 37°C. Glycine buffer (50 μl) were added into each well and measured at 405 nm with reference wave length at 620 nm. In pilot experiments we tested different doses of NMB as well as the NMBR antagonist in murine keratinocytes over days. We performed viability assays and observed a loss of viability with higher antagonist concentrations. Therefore, the antagonist was the limiting factor to the NMB dose in mast cells. For the DRG the neurons were only exposed to NMB for a short time and we did not observe a loss of functionality. Therefore, we were able to increase the NMB dose.

### RNA isolation, complementary DNA transcription and real time RT-PCR

The expression of the NMBR (NM_008703.2) was determined using real time PCR. The Qiagen RNeasy Mini-Kit (RNA isolation) and Qiagen QuantiTect Reverse Transcription Kit (cDNA transcription) were used in accordance with the manufacturer’s instructions (Qiagen, Germantown, MD). Primer were designed and ordered from Sigma. NMBR forward primer (5’-acctctcctttcccacagagg-3’) and NMBR revers primer (5’-atcacacagcggatcaccaa-3’) with a product size of 115 bp was designed. As housekeeping gene, glyceraldehyde-3-phosphate dehydrogenase (GAPDH, NM_008084.3) was chosen (forward primer 5’-cgtcccgtagacaaaatggt-3’, revers primer 5’-gaatttgccgtgagtggagt-3’, product size 177 bp). The following PCR settings were used. An initial activation step of 10 min at 95°C was followed by 3-step cycling (40 cycles): denaturation for 15 s at 95°C, annealing for 30 s at 60°C, and extension for 30 s at 72°C. Melting curve analysis was performed from 55°C to 95°C. Agarose gel electrophoresis was performed with 2% agarose in 1× Tris-Borat-EDTA buffer to visualize the amplification products using the ChemiDoc TM (Bio-Rad, CA).

### Statistical evaluation

Results are presented as single values with median or as paired values. Values in the text are given as median and range. Non-parametric analysis was performed for all data sets based on not normal distribution. Statistical tests applied are indicated in the figure legends. For calcium imaging, comparison of proportions were made using Fisher exact test. GraphPad Prism version 7.0b was used and *p*-values below 5% probability were considered statistically significant.

### Reagents

NMB (Bachem, Bubendorf, Switzerland, H-3280 or from ICN Biomedicals Inc., Aurora, OH; H-Gly-Asn-Leu-Trp-Ala-Thr-Gly-His-Phe-Met-NH2 trifluoroacetate salt, MW 1132.31) was dissolved in sterile phosphate buffered saline (PBS) or saline to the final concentrations as indicated in methods. The NMBR antagonist PD168368 (Santa Cruz Biotechnology, Dallas, TX) was dissolved in DMSO 10 mg/ml and further diluted in vehicle.

## RESULTS

### Neuromedin B induces acute itch in mice and monkeys

**Fig. 1.**
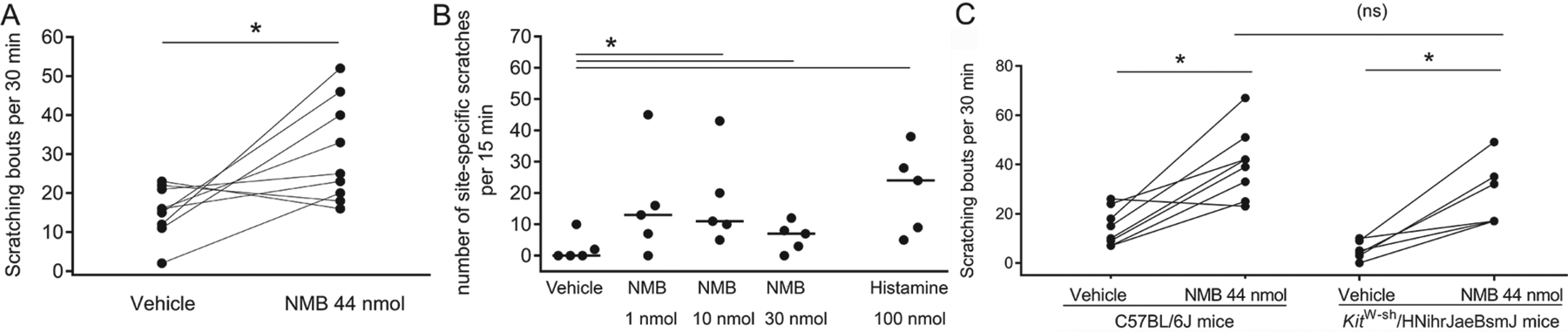
Neuromedin B (NMB) induces acute itch in mice and monkeys and is not mediated via mast cells. **A.** A crossover study with a two-week washout period showed, that intradermal injection of NMB (44 nmol/50 μl) significantly increased scratching bouts with 25 (16–52) in BALB/c mice compared to vehicle control (phosphate buffered saline (PBS) 50 μl) 16 (2–23) within 30 min. *p < 0.05, Mann-Whitney test, single values, n = 9 females group. B. A crossover study with one-week washout period showed, that intradermal injection of NMB (1, 10 and 30 nmol/50 μl) as well as histamine (100 nmol/50 μl) significantly increased scratching bouts in monkeys with 13 (0–45), 11 (5–43) and 7 (0–12) and 24 (5–38), respectively compared to vehicle control (saline 50 μl) with 0 (0–10) within 15 min. *p < 0.05, paired *t*-test, one-tailed, single values and median, n = 5 per group, 1 male and 4 females. C. A crossover study with a two-week washout period in C57BL/6J mice compared to Kit^W-sh^/HNihrJaeBsmJ mice showed, that NMB (44 nmol/50 μl) induced itch in both strains (41 (23–67) versus 25 (17–29) respectively) compared to the vehicle control (saline 50 μl) (13 (7–26) versus 5 (0–10), respectively). Comparison of the treatment groups revealed, that there is no difference between NMB-induced itch between the strains. *p < 0.05, Wilcoxon test, (ns) Mann-Whitney test, single values, *n* = 6–8 per group, 5 females, 3 males.

NMB induced itch when injected intradermally in female BALB/c mice. The 44 nmol/50 μl (50 μg/50 μl) dose was based on what had been reported previously (9), and it had been verified beforehand in a vehicle-controlled pilot experiment with 5 females BALB/c mice per group (data not shown). In the crossover experiment, NMB doubled the number of scratching bouts within 30 min from a median of 16 (range 2–23) bouts in the vehicle group to a median of 25 (range 16–52) bouts in the NMB group (Fig. 1A). The same increase of acute itch was observed in a one-week crossover study in monkeys. Within 15 min, monkeys scratched about twice as much after NMB injection compared to vehicle injection. All NMB concentrations (1 and 10 and 30 nmol/50 μl) led to a median of 13 (range 0–45), 11 (range 5–43), and 7 (range 3–12) scratching bouts, respectively compared to 0 (range 0–10) bouts after vehicle injection (Fig. 1B). In summary, intradermal injections of NMB induce acute itch in two evolutionary-distant species: mice and monkeys.

### Neuromedin B-induced acute itch in mice is not mediated via mast cells

Intradermal injection of NMB (44 nmol/50 μl) significantly induced itch in both C57BL/6J and mast cell-deficient mice Kit^W-sh^/HNihrJaeBsmJ mice (Fig. 1C). In C57BL/6J mice, NMB injection led to a 3 times increase of scratching bouts (23–67) compared to the vehicle (7–26). In Kit^W-sh^/HNihrJaeBsmJ mice, NMB injection even led to a 6x higher scratching rate (17–49) compared to vehicle (0–10). Comparison of the NMB-treated groups revealed a lack of difference in NMB-induced scratching between wild-type and mast cell deficient mice. These observation suggest that the NMB-induced itch in mice does not involve dermal mast cells.

### Allergic itch and inflammation in mice is not augmented through neuromedin B

**Fig. 2.**
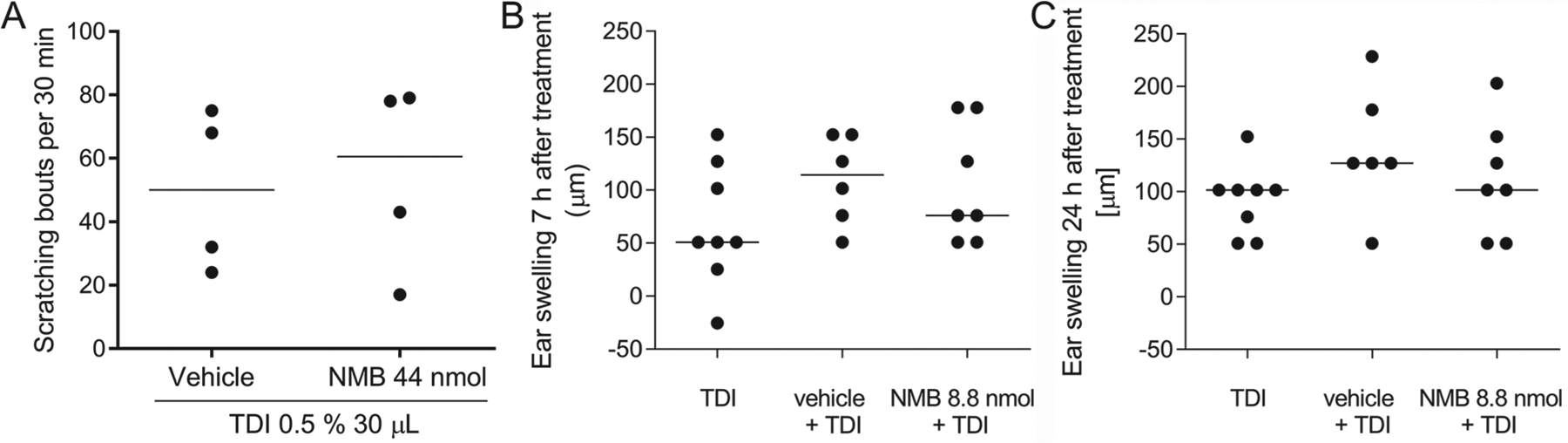
Allergic itch and inflammation in mice is not augmented through neuromedin B (NMB). Mice were sensitized with toluene-diisocyanate (TDI) for 21 days and challenged to test enhancement of itch in the neck and inflammation in the ear on day 28. A. In the allergic dermatitis model in male BALB/c mice, intradermal injection of NMB (44 nmol/50 μl) did not enhance scratching bouts (61 (17–79)) compared to vehicle control (phosphate buffered saline (PBS) 50 μl) (50 (24–75) within 30 min. Mann-Whitney test, single values, *n* = 4 per group. B/C. To test pro-inflammatory effects of NMB (8.8 nmol/10 μl), it was injected intradermally into the ear skin of female BALB/c mice. There was no difference in ear swelling between the vehicle treated group and the NMB treated group after 7 h (114 μm (51–153) and 76 μm (51–178), respectively) and 24 h after injection (127 μm (51–229) and 102 μm (51–203), respectively). Mann-Whitney test, single values with median, *n* = 6–7 per group.

In the TDI model for allergic itch, the mice did not show an increase in scratching bouts after intradermal NMB injection compared to those of the control group. Both vehicle- and NMB-treated mice (8.8 nmol/10 μl) showed a median number of scratching bouts of 50 (range 24–75) and 60 (range 17–79) in the first 30 min after injection (Fig. 2A). Therefore, NMB does not seem to potentiate a classic hapten-induced allergic itch in mice. To test for the pro-inflammatory potential of NMB, we tested it in irritant and allergic settings. In the irritant setting, the intradermal injection of NMB into the ears (8.8 nmol/10 μl) did not induce ear swelling compared to the vehicle-treated animals at various time points over 48 h (data not shown). In the allergic TDI model, ear swelling was determined 7 and 24 h after NMB injection. The NMB-injected ear skin with 76 μm (range 51–178) did no show increased ear swelling 7 h after injection compared to the vehicle treated ear skin with 114 μm (range 51–153) (Fig. 2B). After 24 h, ear swelling was about 102 μm (range 51–203) in the NMB group compared to the vehicle group with 127 μm (range 51–229) (Fig. 2C). Altogether, we concluded that NMB did not induce inflammation in our acute inflammation and allergic model; it also did not enhance allergic itch in the latter.

### Murine dorsal root ganglia express the NMBR and get activated through neuromedin B

**Fig. 3.**
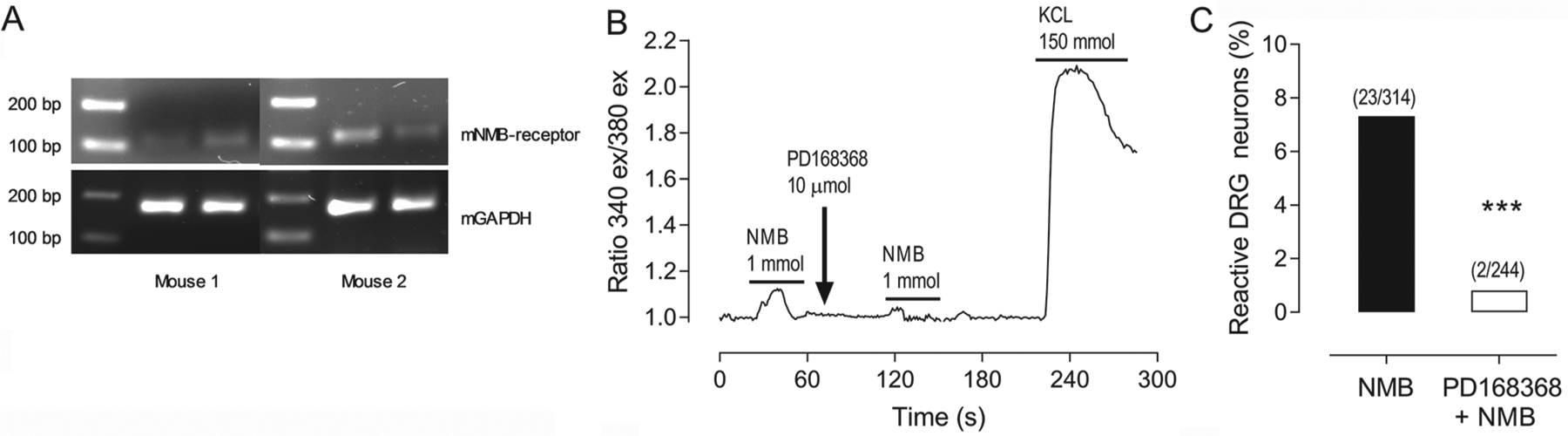
Murine dorsal root ganglia (DRG) express the NMBR and get activated through neuromedin B (NMB). A) Representative 2% agarose electrophoresis gel showing results from 2 out of 4 female BALB/c mice in technical duplicates. The murine NMBR shows specific bands at 115 bp, murine glyceraldehyde-3-phosphate dehydrogenase (GAPDH) bands at 156 bp. B) A representative single cell calcium imaging experiment shows a neuron, that reacts to NMB (1 mM) with an increase of the ratio (340ex/380ex). After the ratio returned to baseline, the NMBR antagonist PD168368 (10 μM) was applied. About 60 s later, repeated stimulation with NMB (1 mM) did not show an increase in the extinction ratio. At the end of the experiment, KCL (150 mM) was applied as the positive control for viable cells. C) Final single cell calcium imaging analysis revealed, that 7.3% DRG neurons reacted to NMB (1 mM). The reaction to NMB was significantly reduced to 0.8% through pre-incubation with the NMBR antagonist PD168368 (10 μM). ***p < 0.001, Fisher’s exact test, *n* = 3 independent experiments from female BALB/c mice.

Murine DRG were found to express the NMBR (Fig. 3A) and single-cell calcium imaging showed that the neurons reacted to NMB (1 mmol/l) with an influx of intracellular calcium. This quantity was determined as the effective dose in a pilot study (data not shown). When the cells were pre-incubated with the NMBR antagonist PD168368 (10 μmol/l) for 60 s, the re-stimulation with NMB led to a reduced calcium influx (Fig. 3B). Overall, about 7% of DRG neurons (23 activated out of 314 neurons total) reacted to the stimulation with NMB (1 mmol/l); this reaction could be blocked almost completely (2 activated out of 244 neurons total) with the NMBR antagonist (Fig. 3C). These results suggest that NMB directly activates mouse DRG neurons via its specific receptor NMBR.

### Murine bone marrow-derived mast cells express the NMBR but do not degranulate upon neuromedin B stimulation

**Fig. 4.**
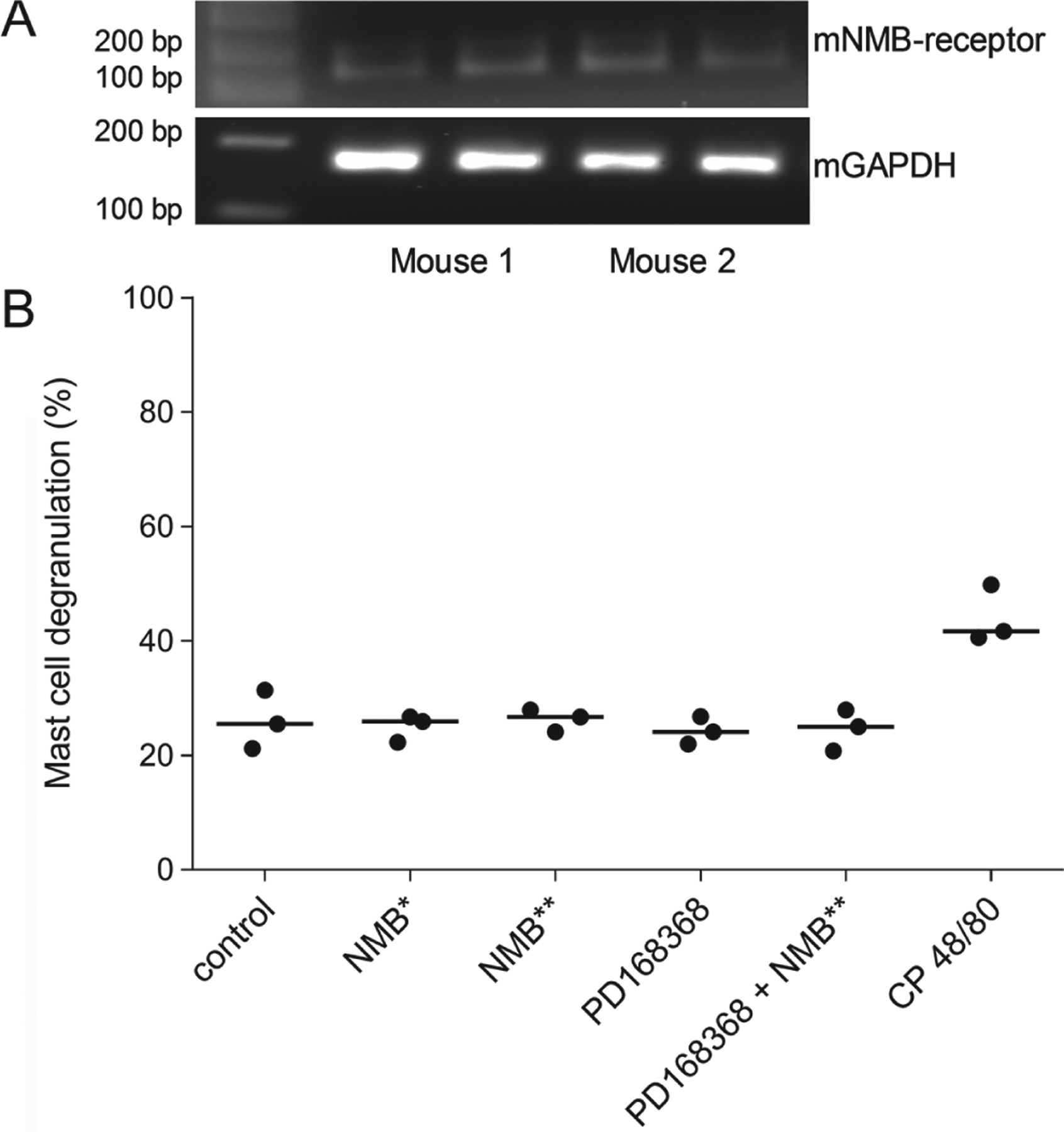
Murine bone marrow-derived mast cells express the NMBR but do not degranulate upon neuromedin B (NMB) stimulation. A. Representative 2% agarose electrophoresis gel showing results from mast cell cultures from two female BALB/c mice in technical duplicates. The murine NMBR shows specific bands at 115 bp and murine glyceraldehyde-3-phosphate dehydrogenase (GAPDH) bands at 156 bp. B. The beta-hexosaminidase assay showed, that murine BMMC did not degranulate upon NMB stimulation (*NMB 0.883 μM (1 μg/ml), **NMB 8.83 μM (10 μg/ml). The NMBR antagonist PD168368 8.83 μM (4.88 μg/ml) and in combination with NMB 8.83 μM (10 μg/ml), also did not induce mast cell granulation compared to the vehicle control. CP48/80 654 μM (100 μg/ml) served as positive control for degranulation. Single values with mean, n = independent experiments from 3 female BALB/c mice.

Murine BMMC expressed the NMBR (Fig. 4A). The beta-hexosaminidase assay showed that NMB (0.883 μM, 8.83 μM) did not increase mast cell degranulation (25 and 26%, respectively) compared to the vehicle control group (26%). The NMBR antagonist PD168368 (8.83 μM) alone, as well as in combination with NMB (8.83 μM), did not induce mast cell degranulation (24 and 25% respectively) (Fig. 4B). In contrast, our positive control compound 48/80 caused mast cell degranulation above 40% (Fig. 2C). We concluded that NMB does not induce the degranulation of murine BMMCs.

## DISCUSSION

In this study, we determined that NMB directly activated murine DRG neurons *in vitro*, and this activation was blocked by a specific NMBR antagonist. *In vivo*, intradermal injections of NMB induced acute itch in mice and monkeys. While it induced itch, it did not potentiate that associated with allergic dermatitis in a hapten-induced Th2-dominant allergic model (16, 19). Finally, we found that NMB did not induce or potentiate dermal inflammation in the acute irritant and allergy model.

NMB is a highly conserved neuromediator among species (7). Herein, we showed that intradermal injections of NMB did not only induce itch in various mouse strains and monkeys, but also in both sexes. It is known, that there are sex differences for itch and pain reception (20). Therefore, we used both male and female mice and NMB induced itch in both sexes. These consistent results emphasize a potentially important evolutionary-conserved role for NMB in acute itch across species.

Both NMB and GRP activate structurally similar but pharmacologically distinct receptors: the NMBR and the GRPR (21, 22). Recently Wan et al. (23) tested the pruritogenic potential of NMB and GRP in the CNS after intrathecal injection. They showed that the itch transmission via their receptors is largely non-overlapping and that they do not compensate for each other in the processing of nociception (23). These investigators used knockout mice with ablation of NMBR neurons using diphtheria toxin cassettes. Knockout models are valuable tools in research and testing NMB as dermal pruritogen in NMB and NMBR knockout mice could support our findings.

For *in vitro* experiments, we used PD168368 as a specific antagonist. The selectivity for PD168368 against the NMBR is described to be 30- to 60-fold compared to the GRPR (human/rat) (24). This was supported by Su & Ko (9), who characterized PD168368 as a specific and potent NMBR antagonist, with no action on the GRPR. We have been hesitant to test PD168368 also *in vivo*, as the data for systemic in vivo use are very scarce (25). Nevertheless, for future studies, we wish to test different NMBR antagonists *in vivo* to inhibit itch pharmacologically.

Our study confirmed the findings by Mishra et al. (10) that murine DRG express a functional NMBR. We could also show that the NMBR antagonist PD168368 almost completely blocks the NMB-induced neuronal activation, thereby indicating the presence of a specific NMBR-mediated signaling. For future studies, it would be interesting to characterize these NMB-responding neurons further, and to determine their reaction towards histamine or chloroquine as well as their expression of CGRP or TRPV1 (10).

In a next step we wanted to elucidate possible involvement of mast cells in NMB action. The bombesin GRP has been shown to induce itch through mast cell degranulation (26). However, our results show that this is unlikely to be the case for NMB. We established herein that murine BMMC express the NMBR, but its function is likely not to induce mast cell degranulation. As the NMBR is present on mast cells, studies will need to address possible function of NMBR on these cells. Corroborating the lack of mast cell degranulation, we found that mast cell deficient mice scratched as much as wild type mice after NMB injection, which is different from what is seen with GRP (26). The different effect of GRP and NMB on mast cell degranulation is a noteworthy yet perplexing observation, suggesting that these two bombesins have distinct roles not only in the transmission of itch in the CNS (23) but our study also indicates different roles in the peripheral signaling cascade.

In the first part of the study, we tested the potential of NMB to induce acute itch. In the second part, we showed that it did not have a potentiating effect on itch and inflammation in an allergic dermatitis model. A possible explanation could be that the levels of NMB in the skin of patients with allergic-inflammatory pruritus are already high and the receptor is already activated. This would be in line with our findings of a 2–3 fold increase of NMB mRNA expression in acute AD lesions that follow an epicutaneous allergen challenge in sensitized dogs (13). Because we did not see an increase in itch in the TDI model, we did not look for increased NMB levels in skin lesions. Nevertheless, future studies should characterize the role of NMB in chronic allergic skin lesions. To assess the potential of NMB to induce local inflammation, we used an irritative as well as an allergic dermatitis model. NMB did not induce inflammation in either setting. This is in contrast to the results from Mishra et al. in 2012 (10), in which intraplantar injections of NMB resulted in neurogenic inflammation. The applied volume and NMB concentration were the same in both studies. A possible explanation for the different outcome could be the site of application (ear vs paw) and the route of administration (intradermal vs. intraplantar). Andoh et al. (26) could trigger itch when GRP was injected intradermally and they related this to the degranulation of mast cells. To our knowledge, our study is the first to test NMB as a potential dermal pruritogen.

There are some limitations of the study. First, we were only testing for itch induction and the rostral back model is a well-established model for testing this sensation. The cheek model is a valuable tool to distinguish between itch and pain. As shown by Akiyama et al. in 2010 (27) the amount of hind-limb scratching is similar between the rostral back and cheek models in mice and rats. We also did not see any pain-related behavior when NMB was injected intradermally in monkeys. Future studies should also determine any dose dependency existing in each mouse strain and sex. To confirm our observations, NMBR and GRPR-knockout mice would be a valuable tool to distinguish between NMB and GRP-induced itch. With the identification of NMB as yet another dermal pruritogen, our study contributes an additional piece of the itch pathogenesis puzzle. We hypothesize that such itch induction is due to the NMB directly binding to the NMBR on sensory neurons. The bombesin NMB may be an additional target to investigate for the development of novel mechanism-based strategies to treat itch.

In comparison to acute itch, stimulus-specific patterns of activation are changed in diseases where chronic itch is involved. But to understand these changes during chronic itch, more studies are necessary. This study provides a first attempt to characterize the pruritogenic potential of NMB in the skin. With the background known so far the pathogenesis of pruritus is quite complex. It remains important to investigate and subsequently develop novel mechanism-based strategies to treat pruritus and possibly provide a locus for pharmacological control of pruritus.

## SIGNIFICANCE

Neuromedin B is expressed in neurons involved in itch but its peripheral pruritogenic potential is not well described. Neuromedin B induces itch in mice and monkeys when injected into the skin. Most likely this itch signal is transmitted through the activation of peripheral neurons. So far the pathogenesis of pruritus is quite complex. It remains important to investigate and subsequently develop novel mechanism-based strategies to treat pruritus and possibly provide a locus for pharmacological control of pruritus. Thus, Neuromedin B could be an interesting new therapeutic target for peripheral processing of itch at the level of sensory neurons.
